# Supplementary material for: Further Insights into Invasion: Field Observations of Behavioural Interactions between an Invasive and Critically Endangered Freshwater Crayfish Using Baited Remote Underwater Video (BRUV)
Source: Biology (Basel). 2022 Dec 22;12(1):18. doi: 10.3390/biology12010018 (PMC9855398; doi:10.3390/biology12010018)
Supplement: Supplementary file 1 [file biology-12-00018-s001.zip › Supplementary figure S1.pdf]

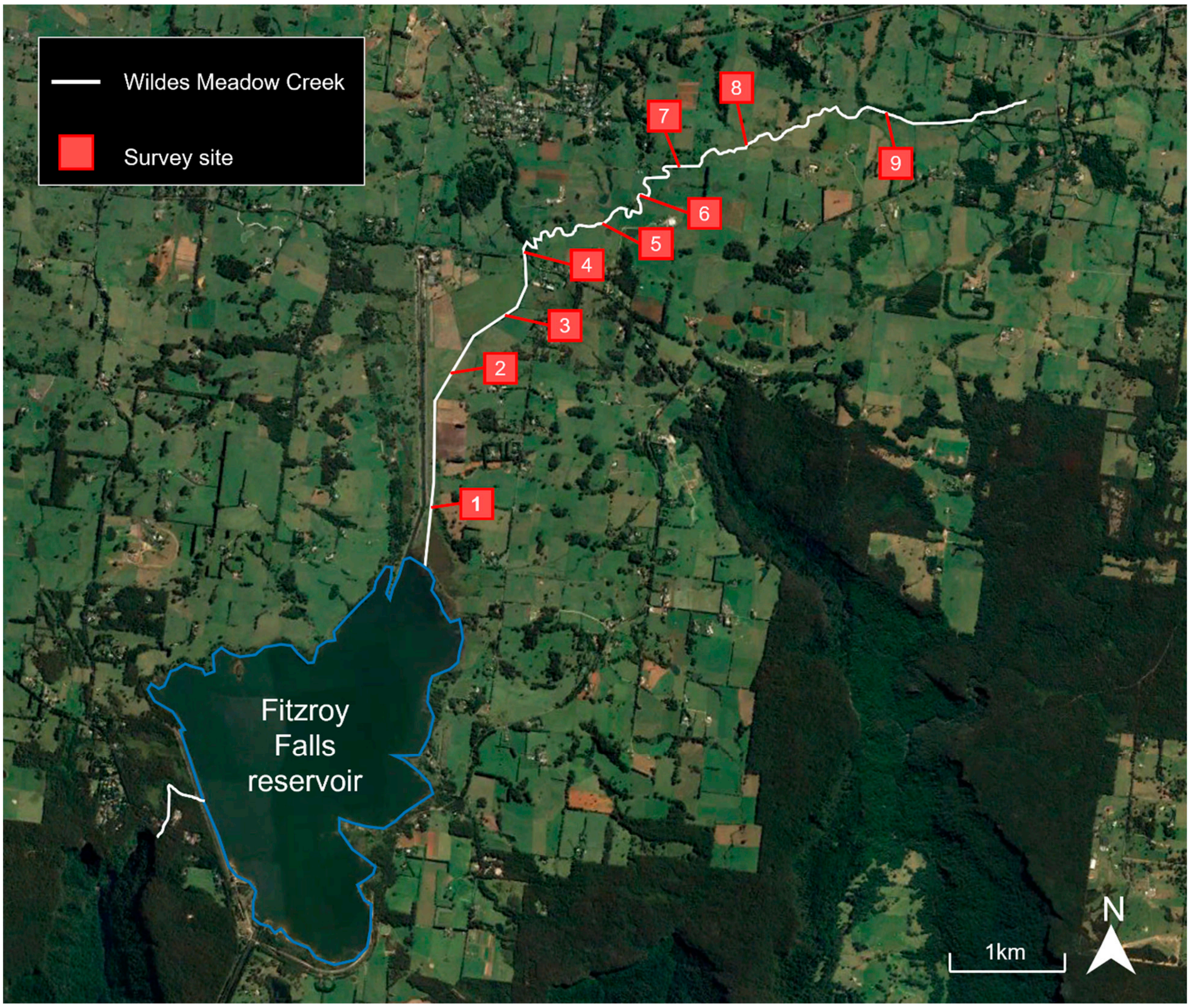

Supplementary figure S1. Map indicating Wildes Meadow Creek and the positions of the nine selected survey sites.
